# Predicting language outcome at birth

**DOI:** 10.3389/fnhum.2024.1370572

**Published:** 2024-07-05

**Authors:** Maria Clemencia Ortiz-Barajas

**Affiliations:** CNRS, IKER (URM 5478), Bayonne, France

**Keywords:** EEG, theta activity, newborns, language development, predictability

## Abstract

Even though most children acquire language effortlessly, not all do. Nowadays, language disorders are difficult to diagnose before 3–4 years of age, because diagnosis relies on behavioral criteria difficult to obtain early in life. Using electroencephalography, I investigated whether differences in newborns’ neural activity when listening to sentences in their native language (French) and a rhythmically different unfamiliar language (English) relate to measures of later language development at 12 and 18 months. Here I show that activation differences in the theta band at birth predict language comprehension abilities at 12 and 18 months. These findings suggest that a neural measure of language discrimination at birth could be used in the early identification of infants at risk of developmental language disorders.

## 1 Introduction

Most children acquire their native language(s) rapidly and effortlessly during the first years of life regardless of culture (Kuhl, 2004). However, this is not always the case. Around 7% of kindergarten children (5–6 years) (Tomblin et al., 1997) are identified as having specific language impairment (SLI, also known as developmental language disorder, DLD), a disorder characterized by the difficulty to understand and produce spoken language in the absence of other cognitive deficits. Another 5 to 17% of school children suffer from dyslexia (Shaywitz, 1998), a specific deficit in reading acquisition not attributable to low IQ, poor education or neurological damage (Ramus and Ahissar, 2012). If untreated, these disorders can have an impact on many aspects of the child’s life (social, behavioral, academic), which can persist until adulthood. Nowadays, language disorders are difficult to diagnose before 3–4 years of age (Cristia et al., 2014), because diagnosis relies on behavioral criteria that are difficult to obtain early in life. However, children with learning or reading disabilities typically show deficits in speech perception earlier than when their disorder is diagnosed (Kuhl et al., 2005). Identifying measures that could allow their earlier detection is fundamental for the design of earlier interventions.

Previous research has shown that phonological deficits are often found in individuals with dyslexia and/or SLI (Ramus, 2003; Schulte-Körne and Bruder, 2010; Leonard, 2014). However, whether these deficits are speech-specific or related to basic auditory perception is still under debate (Lorusso et al., 2014; Cantiani et al., 2016). Furthermore, deficits processing auditory information in early infancy/childhood have been shown to relate to poorer later language and literacy skills in school (Molfese, 2000; Leppänen et al., 2010; Van Zuijen et al., 2013; Schaadt et al., 2015; Cantiani et al., 2016; Lohvansuu et al., 2018). Molfese (2000) found that the amplitude and latency of ERPs recorded at birth while infants listened to speech and non-speech sounds, could predict with 81% accuracy whether at 8 years of age children would be identified as normal, poor or dyslexic readers. In another newborn study, Leppänen et al. (2010) showed that children with familial risk for dyslexia exhibited atypical processing of sound frequency at birth, as evidenced by their ERP response to tones varying in pitch. Additionally, these early differences in auditory processing were related to phonological skills and letter knowledge before school age, as well as to phoneme duration perception, reading speed and spelling accuracy in the second grade of school (Leppänen et al., 2010). Similarly, Cantiani et al. (2016) investigated Rapid Auditory Processing (RAP) abilities in 6 months-olds at risk for Language Learning Impairment (LLI), by assessing their discrimination of pairs of tones varying in frequency and duration. They found their ERPs to be atypical and to be predictive of their expressive vocabulary at 20 months (Cantiani et al., 2016). More recently, Mittag et al. (2021) used magnetoencephalography (MEG) to investigate auditory processing of white noise in 6 and 12-month-olds. They found atypical auditory responses in infants at risk for dyslexia, which predicted syntactic processing between 18 and 30 months, and as well as word production at 18 and 21 months. However, this predictive relation was not found for the control infants.

Other studies have also investigated whether early speech perception abilities relate to later language acquisition. This is supported by the native language neural commitment (NLNC) hypothesis (Kuhl, 2000, 2004) which proposes that early linguistic experience with the native language produces dedicated neural networks that influence the brain’s ability to learn language. This hypothesis suggests that infants’ early skills in native-language phonetic perception should predict infants’ later language abilities (Kuhl, 2004). Tsao et al. (2004) tested this hypothesis by performing one of the first studies exploring the link between speech perception and language acquisition before the age of 2 years. They used the conditioned head-turn task to test 6-month-old infants on a speech discrimination task (a vowel contrast perceived by adults as native), and found significant correlations between their speech perception skills at 6 months and vocabulary measures (words understood, words produced and phrases understood) at 13, 16 and 24 months. In a follow-up study, Kuhl et al. (2005) tested a similar paradigm on 7 month-olds, this time with two conditions: one contrast from their native language, and one from a non-native language. They found that both native and non-native phonetic perception abilities were related to later measures of language outcome but in opposite directions: better native-language discrimination at 7 months was positively correlated to expressive vocabulary at 18 and 24 months, whereas better non-native-language discrimination was negatively correlated to expressive vocabulary at 18 and 24 months. These findings were supported by an electrophysiological study comparing ERP responses in 11-month-olds to native and foreign speech contrasts (Rivera-Gaxiola et al., 2005). They showed that infants who exhibited larger (more positive) P150-250 amplitudes to the foreign deviant with respect to the standard produced more words at 18, 22, 25, 27, and 30 months, than those who displayed larger (more negative) N250-550 amplitudes to the foreign deviant with respect to the standard, at the same ages. A later ERP study from the same team showed that ERP responses to native and non-native contrasts at 7 months also related to later language outcomes, again in opposing directions: greater negativity of the MMN (mismatch negativity) to native language phonetic contrasts at 7 months was associated with a larger number of words produced at 18 and 24 months, whereas more negative MMNs to non-native language phonetic contrasts at 7 months predicted fewer words produced at 24 months (Kuhl et al., 2008). Kuhl et al. (2008) suggest that increased sensitivity in the perception of native phonetic contrasts is indicative of neural commitment to the native language, whereas sensitivity to non-native contrasts reveals uncommitted neural circuitry. The ERP responses shown in these studies seem to be a reflection of this level of neural commitment, which in turn predicts language scores at later ages (Rivera-Gaxiola et al., 2005; Kuhl et al., 2008).

Previous linguistic studies focused on the discrimination of phonetic contrasts as the early measure of speech perception that could predict later language skills. A recent electroencephalography (EEG) study explored whether neural tracking of sung nursery rhymes during infancy could predict language development in infants with high likelihood of autism (Menn et al., 2022). Autistic children often show delay in language acquisition (Howlin, 2003), which is why identifying measures that could predict later language skills is relevant for this population. Menn et al. (2022) found that infants with higher speech-brain coherence in the stressed syllable rate (1–3 Hz) at 10 months showed higher receptive and productive vocabulary (words understood and words produced) at 24 months, but no relationship with later autism symptoms. They suggest that these results could reflect a relationship between infants’ tracking of stressed syllables and word-segmentation skills (Menn et al., 2022), which in turn predict later vocabulary development (Junge et al., 2012; Kooijman et al., 2013). Similarly, a recent study investigating word learning at birth revealed that neonates can memorize disyllabic words so that having learnt the first syllable they can predict the word ending, and the quality of word-form learning predicts expressive language skills at 2 years (Suppanen et al., 2022).

To my knowledge, most studies investigating infant speech perception abilities as possible predictors of later language development have tested infants using phonetic contrasts (Tsao et al., 2004; Kuhl et al., 2005; Rivera-Gaxiola et al., 2005; Kuhl et al., 2008), bi-syllabic pseudo-words (Suppanen et al., 2022), and nursery rhymes (Menn et al., 2022). However, perception abilities of natural speech have rarely been used as predictors. Here, I explore the potential of using EEG measures at birth in response to naturally spoken sentences in the native language (prenatally heard) and a rhythmically different unfamiliar language as predictors of later language skills in typically-developing infants.

At birth, infants are equipped with a rich set of speech perception abilities that help them acquire language from the get-go. Some of these are universal, broad-based abilities, in place independently of what language they heard *in utero* (Ortiz Barajas and Gervain, 2021). For instance, newborns can recognize speech, and show preference for it over equally complex speech analogs (Vouloumanos and Werker, 2007). They are also able to discriminate two languages, even if they are unfamiliar to them, on the basis of their different rhythms (Mehler et al., 1988; Nazzi et al., 1998; Ramus et al., 2000), but they are unable to discriminate them if their rhythms are similar (Nazzi et al., 1998; Ramus et al., 2000). Interestingly, newborns also exhibit speech perception abilities shaped by prenatal experience with the language(s) spoken by their mother during the last trimester of pregnancy. Newborns’ prenatal experience with speech mainly consists of language prosody, i.e., rhythm and melody, because maternal tissues filter out the higher frequencies, necessary for the identification of individual phonemes, but preserve the low-frequency components that carry prosody (Pujol et al., 1991). On the basis of this experience, newborns are able to recognize their native language, and prefer it over other languages (Mehler et al., 1988; Moon et al., 1993). Furthermore, it has been shown that recognizing the language heard *in utero*, goes beyond simply discriminating it from an unfamiliar one, as monolingual and bilingual newborns exhibit different patterns when presented with the same pair of rhythmically different languages: monolinguals, who are familiar with one of the languages being contrasted, discriminate them, and prefer the familiar language; while bilinguals, who are familiar with both languages being contrasted, discriminate them and show equal preference for both languages (Byers-Heinlein et al., 2010).

Building up on previous research showing that the discrimination of native/non-native phonetic contrasts predicts later language skills (Kuhl et al., 2005; Rivera-Gaxiola et al., 2005; Kuhl et al., 2008), here I explore whether newborns’ ability to discriminate languages on the basis of their different rhythms could relate to language development. It has been suggested that individuals with dyslexia have difficulty extracting stimulus regularities from auditory inputs (Daikhin et al., 2017), therefore a rhythmic discrimination task, which requires detecting regularities in speech rhythm, represents a good predictor candidate for this population.

The neural mechanisms that support rhythmic discrimination in infants are not fully understood (Ortiz Barajas and Gervain, 2021). Previous infant studies have shown that low-frequency neural activity (delta and/or theta band) reflect language discrimination at birth (Ortiz-Barajas et al., 2023) and at 4.5 months (Nacar Garcia et al., 2018). Since rhythm is carried by the low-frequency components of the speech signal (Rosen, 1992), specifically the syllabic rate, it is reasonable for rhythm to be encoded by the low-frequency oscillations delta and theta. In adults, theta activity has been claimed to support the processing of syllables. This claim has mainly been based on two facts: (1) the syllabic rate of speech, roughly 4-5 Hz (Ding et al., 2017; Varnet et al., 2017), corresponds to the frequencies of the theta band (Giraud and Poeppel, 2012), and (2) brain responses in the theta band have been shown to synchronize to the speech envelope, corresponding to the slow overall amplitude fluctuations of the speech signal over time, with peaks occurring roughly at the syllabic rate (Gross et al., 2013; Molinaro et al., 2016; Vander Ghinst et al., 2016; Zoefel and VanRullen, 2016; Pefkou et al., 2017; Song and Iverson, 2018). Furthermore, newborns’ neural activity has been found to track (synchronize to) the speech envelope of familiar and unfamiliar languages equally well, suggesting that envelope tracking at birth represents a basic auditory ability that helps newborns encode the speech rhythm of familiar and unfamiliar languages, supporting language discrimination (Ortiz Barajas et al., 2021; Ortiz Barajas and Gervain, 2021).

**Figure 1:**
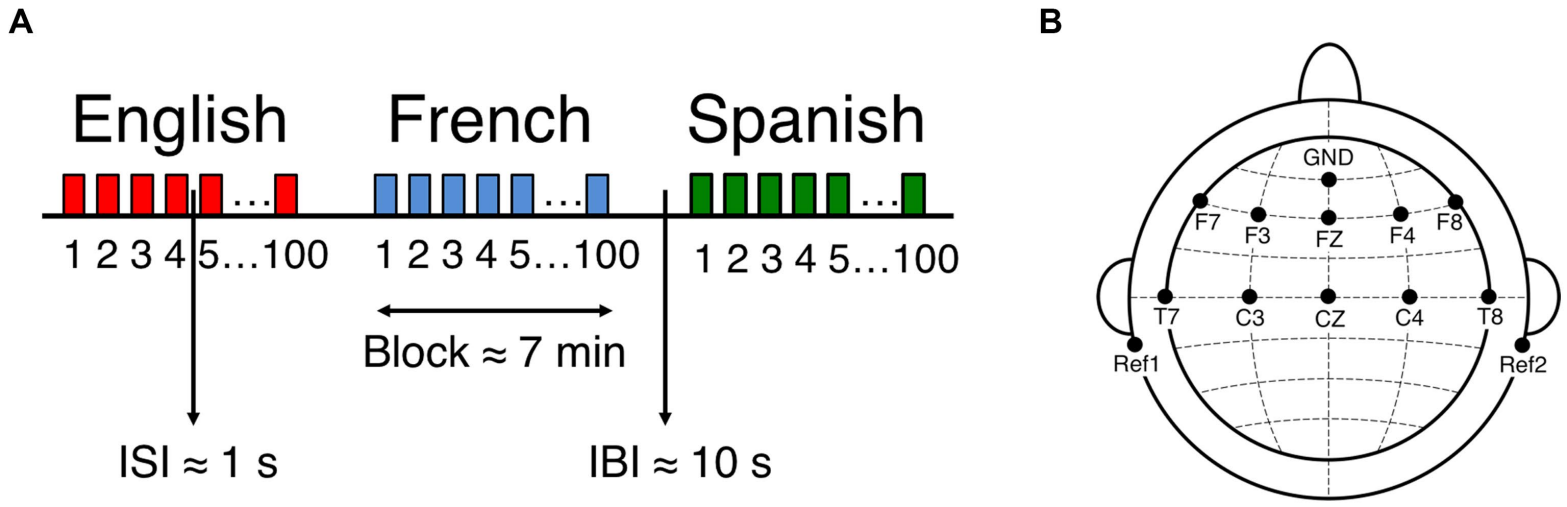
EEG experimental setup and design. **(A)** Experiment block design. ISI: Interstimulus interval, IBI: Interblock interval. **(B)** Location of recorded channels according to the international 10–20 system. Figure adapted from Ortiz Barajas et al. (2021).

To explore the use of a neural measure of language discrimination at birth as a predictor of language outcome, I recorded EEG data from 51 full-term, healthy newborns (mean age: 2.39 days; range: 1–5 days; 20 females), born to French monolingual mothers, while they listened to naturally spoken sentences in three languages: their native language, i.e., the language heard prenatally, French, a rhythmically similar unfamiliar language, Spanish, and a rhythmically different unfamiliar language, English (Figure 1A illustrates the study design). As infants were tested within their first 5 days of life, their experience with speech was mostly prenatal. Based on the above mentioned speech perception abilities, it is reasonable to assume that participants should be able to discriminate and prefer the prenatally heard language French (syllable-timed) from English (stress-timed) based on their different rhythms, but not from Spanish (syllable-timed), as they are rhythmically similar. Given that stimuli are presented in 7 min blocks, and languages are not contrasted closely, I hypothesize that for language recognition to take place, the newborn brain compares each language to the long-term representation it has formed from prenatal experience, in order to recognize familiar features. This hypothesis is supported by one recent study from my team investigating the role of prenatal experience on long-range temporal correlations (LRTC) using a superset of the EEG dataset used here (Mariani et al., 2023), revealing that the newborn brain exhibits stronger correlations in the theta band after being exposed to the native language (French) than to the rhythmically similar (Spanish) and the rhythmically different (English) unfamiliar languages, indicating the early emergence of brain specialization for the native language. These findings support the hypothesis that participants from this study did recognize the prenatally heard language, and that such recognition is reflected by theta activity.

I assessed language rhythmic discrimination at birth as the neural activation difference between the native language (French) and the rhythmically different unfamiliar language (English). I expect this discrimination measure to reflect neural commitment to the native language and in turn to predict language scores at later ages as follows: higher discrimination measures should predict higher language scores, reflecting commitment to the native language, whereas lower discrimination measures should predict lower language scores, reflecting uncommitted neural circuitry. Spanish sentences were presented in this experiment as part of a larger project investigating speech perception at birth. However, here I do not present results for Spanish, as I focus on the rhythmic discrimination of the native language (French) and the rhythmically different unfamiliar language (English).

**Figure 2:**
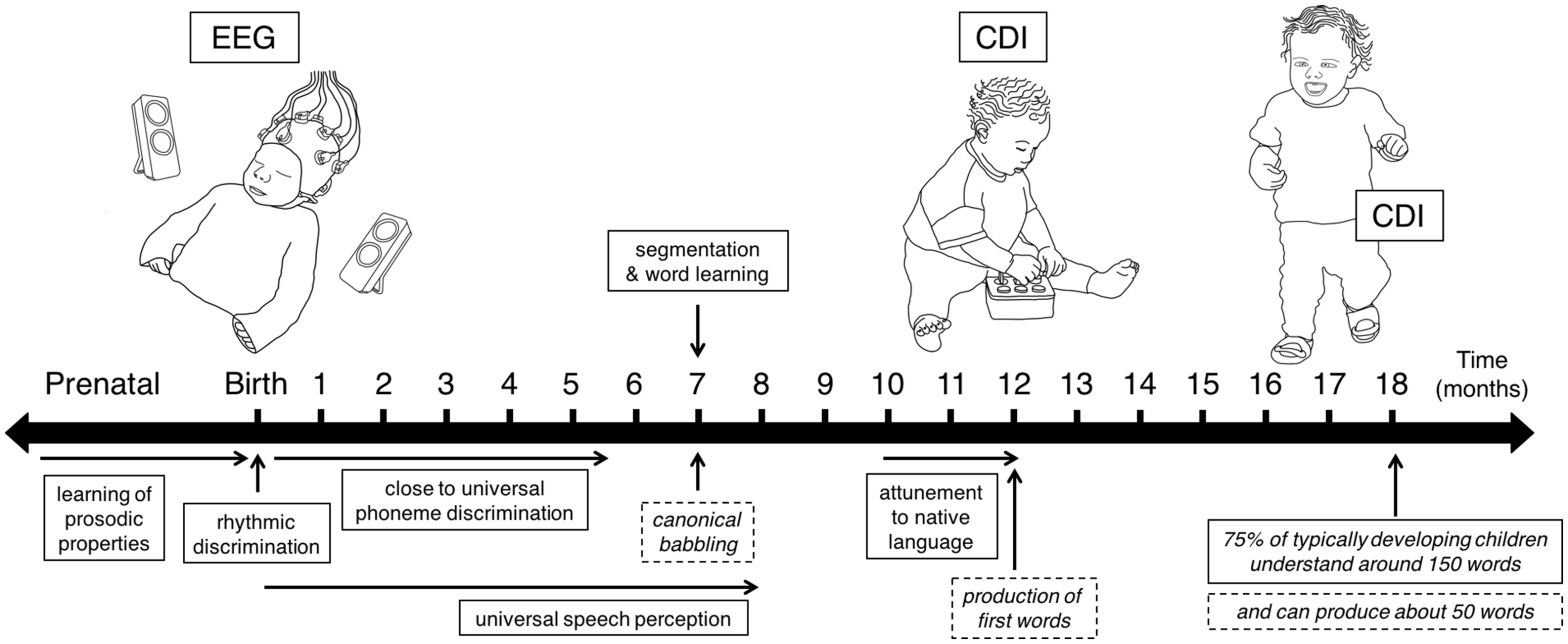
Study timeline indicating the time points when longitudinal data were collected, and displaying some of the developing speech perception (solid boxes) and production (dashed boxes) abilities children exhibit during the first 18 months of life.

To explore the potential use of this neural discrimination measure as a predictor of language outcome, participants were followed longitudinally in order to describe their developmental trajectory, and to look at their individual variability. Figure 2 displays the timeline of the longitudinal study: EEG data were recorded at birth, followed by the collection of information about the participants’ vocabulary size at 12 and 18 months using the MacArthur-Bates Communicative Developmental Inventory (CDI) questionnaires. The participants’ receptive and expressive vocabulary sizes were estimated from the CDI questionnaires, in order to track their language development, and relate it to their neural measures at birth. To assess the predictive role of language discrimination at birth on later language abilities, I conducted a path analysis including newborns’ performance at discriminating the native language (French) from a rhythmically different unfamiliar one (English), and their measures of vocabulary size at 12 and 18 months (number of words understood and number of words produced). A total of 51 infants contributed neural data at birth, and 35 of them contributed with at least one CDI questionnaire at the subsequent ages. Vocabulary data were collected from 27 participants at 12 months, and 30 participants at 18 months (Supplementary Table S1).

## 2 Materials and methods

The EEG data from this study were acquired as part of a larger project that aimed to investigate speech perception during the first two years of life. One previous publication presented a superset of the current dataset (47 participants) evaluating speech envelope tracking in newborns and 6-month-olds (Ortiz Barajas et al., 2021). A second publication, evaluating the role of neural oscillations during speech processing at birth, presented a subset (40 participants) of the initial publication (Ortiz-Barajas et al., 2023). A third publication, exploring changes in neural dynamics at birth, presented a subset (33 participants) of the initial publication (Mariani et al., 2023). These three publications evaluated different hypotheses, therefore analyzing different aspects of the data, which explains the differences in sample size. The EEG dataset used in this manuscript (29 participants) represents a subset of that used in the previous publications, as not all participants contributed with vocabulary measures at 12 and 18 months The processed EEG data that support the findings of this study have been deposited in the OSF repository https://osf.io/4w69p.

### 2.1 Participants

The protocol for this study was approved by the CER Paris Descartes ethics committee of the Paris Descartes University (currently, Université Paris Cité). All parents gave written informed consent prior to participation, and were present during the testing session.

For the first measure of the study, newborns were recruited at the maternity ward of the Robert-Debré Hospital in Paris, where they were tested during their hospital stay. The inclusion criteria were: (i) being full-term and healthy, (ii) having a birth weight > 2,800 g, (iii) having an Apgar score > 8, (iv) being maximum 5 days old, and (v) being born to French native speaker mothers who spoke this language at least 80% of the time during the last trimester of the pregnancy according to self-report. A total of 54 newborns took part in the EEG experiment. However, 3 participants failed to complete the recording due to fussiness and crying (*n* = 2), or technical problems (*n* = 1); and were thus excluded from the longitudinal study. The remaining **51 newborns** (20 girls, 31 boys; age 2.39 ± 1.17 d; range 1–5 d) were followed longitudinally by means of the CDI questionnaires.

For the second and third measures of the study, parents of the infants who contributed with EEG data at birth were requested to fill out vocabulary questionnaires when their children turned 12 and 18 months. As it is often the case in longitudinal studies, some of the participants did not contribute measures to all the assessments. A total of **35 participants** contributed at least one vocabulary questionnaire (at 12 and/or 18 months), of which 27 participants contributed CDI data at 12 months, and 30 participants at 18 months. Supplementary Table S1 presents the list of participants and the data points that they contributed longitudinally.

From the 35 participants who contributed EEG recordings and vocabulary data, 6 participants were excluded due to bad EEG data quality in at least one of the language conditions of interest (French and English). Therefore, a final sample of **29 participants** contributed good quality EEG data at birth, and were included in the prediction analyses: a subset of **22 participants** contributed CDI data at 12 months, while a subset of **27 participants** contributed CDI data at 18 months.

### 2.2 Procedure

Figure 2 presents a timeline highlighting the three ages when data were collected: EEG data at birth, and CDI data at 12 months and 18 months.

For the first measure of the study, newborns were presented with naturally spoken sentences in three languages while their neural activity was simultaneously recorded using EEG. The recording sessions were conducted in a dimmed, quiet room at the Robert-Debré Hospital in Paris, while newborns were comfortably asleep or at rest in their hospital bassinets. The stimuli were delivered bilaterally through two loudspeakers positioned on each side of the bassinet (Figure 2, EEG recording at birth) using the experimental software E-Prime. The sound volume was set to a comfortable conversational level (~65–70 dB). Participants were divided into 3 groups, where each group heard a different set of sentences: 17 newborns heard set1, 17 newborns heard set2, and 17 newborns heard set3. Supplementary Table S2 presents the three sets of sentences used in the study. Participants were presented with one sentence per language (French, English, Spanish), which was repeated 100 times to ensure sufficiently good data quality. The experiment consisted of 3 blocks, each block containing the 100 repetitions of the test sentence in a given language, each block thus lasted around 7 min. An interstimulus interval of random duration (between 1 and 1.5 s) was introduced between sentence repetitions, and an interblock interval of 10 s was introduced between language blocks (Figure 1A). The order of the languages was pseudo-randomized and approximately counterbalanced across participants. The entire recording session lasted about 21 min.

For the second and third measures of the study, parents were requested to fill out the French version of the MacArthur-Bates Communicative Developmental Inventory (CDI) questionnaires (Kern, 2007) when their child turned 12 and 18 months. In each case they were asked to return the questionnaire before their child turned 13 and 19 months respectively, to ensure that the measurement would not exceed these age limits. In order to make it easier for parents to complete the questionnaires, I provided them with the short version of the CDI, which is one page long. The short version CDI has been shown to be as reliable as the original version for the English CDI (Floccia et al., 2018). For the measurement at 12 months I used the *Words and Gestures* CDI, which inquires about the child’s babbling skills, provides a list of 83 words for parents to indicate whether the child understands them and spontaneously produces them, and a list of 25 gestures for them to indicate if the child makes them (e.g., shake the head to say no). For the measurement at 18 months I used the *Words and Sentences* CDI, which provides a list of 97 words for parents to indicate whether the child understands them and spontaneously produces them, and inquires whether the child has started to combine words together.

### 2.3 Stimuli

At birth, I presented infants with sentences in three languages: their native language (French), a rhythmically similar unfamiliar language (Spanish), and a rhythmically different unfamiliar language (English). The stimuli consisted of sentences taken from the story Goldilocks and the Three Bears. Sentences were divided in three sets, where each set comprised the translation of a single utterance into the 3 languages (English, French and Spanish). For instance, set 1 contained the following three sentences: *The bears lived all together in a beautiful house* (English); *Les ours habitaient tous ensemble dans une maison* (French); *Los osos vivían juntos en una casa* (Spanish). The translations were slightly modified by adding or removing adjectives (or phrases) from certain sentences in order to match the duration and syllable count across languages within the same set. All sentences were recorded in mild infant-directed speech by a female native speaker of each language (a different speaker for each language), at a sampling rate of 44.1 kHz. There were no significant differences between the sentences in the three languages in terms of minimum and maximum pitch, pitch range and average pitch. Supplementary Table S2 presents detailed information about the 9 sentences used as stimuli (i.e., duration, syllable count, pitch), and Supplementary Figures S1, S2 display the sentences’ time-series, and frequency spectra, respectively. Additionally, the amplitude and frequency modulation spectra as defined by Varnet et al. (2017) are presented in the Supplementary Figure S3. Utterances were found to be similar in every spectral decomposition. The intensity of all recordings was adjusted to 77 dB.

### 2.4 EEG data acquisition

EEG data were recorded at birth with active electrodes and an acquisition system from Brain Products (actiCAP & actiCHamp, Brain Products GmbH, Gilching, Germany). A 10-channel layout was used to acquire cortical responses from the following scalp positions: F7, F3, FZ, F4, F8, T7, C3, CZ, C4, T8 (Figure 1B). These recording locations were chosen in order to include those where auditory and speech perception related neural responses are typically observed in infants (Stefanics et al., 2009; Tóth et al., 2017) (channels T7 and T8 used to be called T3 and T4 respectively). An additional electrode was placed on each mastoid for online reference, and a ground electrode was placed on the forehead. Data were referenced online to the average of the two mastoid channels, and they were not re-referenced offline. Data were recorded at a sampling rate of 500 Hz, and online filtered with a high cutoff filter at 200 Hz, a low cutoff filter at 0.01 Hz and an 8 kHz (−3 dB) anti-aliasing filter. The electrode impedances were kept below 140 kΩ.

### 2.5 EEG data analysis

The EEG data were processed using custom Matlab® scripts. To extract the low-frequency activity of interest (delta and theta), the continuous EEG signals were band-pass filtered between 1 and 8 Hz with a zero phase-shift Chebyshev filter. The filtered signals were then segmented into a series of 2,560-ms long epochs. Each epoch started 400 ms before the utterance onset (corresponding to the pre-stimulus baseline), and contained a 2,160 ms long post-stimulus interval (corresponding to the duration of the shortest sentence). All epochs were submitted to a three-stage rejection process to exclude the contaminated ones: (1) Epochs with peak-to-peak amplitude exceeding 150 μV were rejected. (2) Epochs with a standard deviation (SD) higher than 3 times the mean SD of all non-rejected epochs, or lower than one-third the mean SD were rejected. (3) The remaining epochs were visually inspected to remove any residual artifacts. Participants who had less than 20 remaining epochs in a given condition after epoch rejection were excluded. From the 35 participants who contributed EEG and CDI data, 6 were excluded due to bad data quality resulting in an insufficient number of non-rejected epochs in one of the language conditions of interest (French and English). Therefore, 29 participants contributed good quality EEG data for the French and English conditions (Supplementary Table S1). The included participants contributed on average 41 epochs (SD: 13.14; range: 20–79) for French, and 35 epochs (SD: 10.69; range: 20–62) for English. The number of non-rejected epochs from the 29 participants were submitted to a paired samples t-test (two-tail), and it yielded no significant differences between the two language conditions [*p* = 0.082].

The non-rejected epochs were subjected to time-frequency analysis to uncover stimulus-evoked oscillatory responses using the Matlab® toolbox ‘WTools’ (Parise and Csibra, 2013). With this toolbox, a continuous wavelet transform of each non-rejected epoch was performed using Morlet wavelets (number of cycles 3.5) at 1 Hz intervals in the 1–8 Hz range. The full pipeline is described in detail in (Csibra et al., 2000; Parise and Csibra, 2013). Briefly, complex Morlet Wavelets are computed at steps of 1 Hz with a sigma of 3.5. The real and the imaginary parts of the wavelets are computed separately as cos and sin components, respectively. The signal is then convoluted with each wavelet. The absolute value of each complex coefficient is then computed. This process resulted in a time-frequency map of spectral amplitude values (not power) per epoch.

**Figure 3:**
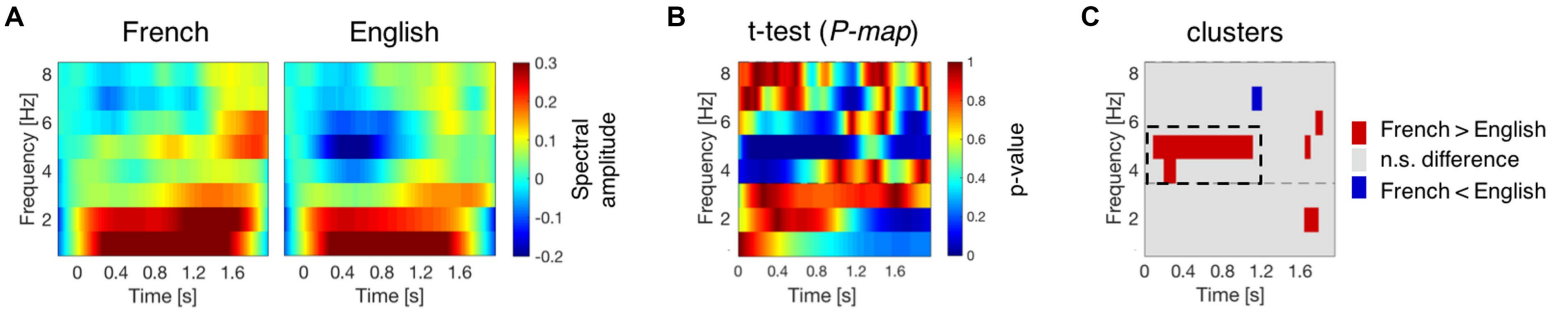
Neural activation during speech processing at birth. **(A)** Average time-frequency response to French and English at channel F4. The time-frequency maps illustrate the mean spectral amplitude per condition from 1 to 8 Hz. The color bar to the right of the figure shows the spectral amplitude scale of the maps. **(B)** P-map obtained by submitting the time-frequency responses to French and English to paired-samples t-tests (two-tailed). **(C)** Time-frequency regions where the absolute *T*-values exceed the critical threshold (|*T*-value| > 2.048). Red regions indicate higher activation for French, while blue regions indicate higher activation for English. The dashed rectangular box indicates the cluster exhibiting significant differences between French and English at channel F4.

Time-frequency transformed epochs were then averaged for French and English separately. To remove the distortion introduced by the wavelet transform, the first and last 200 ms of the averaged epochs were removed, resulting in 2,160 ms long segments, including 200 ms before and 1,960 ms after stimulus onset. The averaged epochs were then baseline corrected using the mean amplitude of the 200 ms pre-stimulus window as baseline, subtracting it from the whole epoch at each frequency. This process resulted in a time-frequency map of spectral amplitude values per condition and channel, at the participant level. The group mean (29 participants) of these time-frequency maps for channel F4 is presented in Figure 3A as an example.

Language discrimination between French (the native language) and English (the rhythmically different unfamiliar language) was assessed by submitting the spectral amplitude values from their time-frequency responses to paired-samples t-tests (two-tailed). Figure 3B displays the *P*-map for this analysis in channel F4, and Figure 3C highlights the time-frequency regions where the absolute *T*-values for this comparison exceed the critical threshold (|*T*-value| > 2.048). Cluster-level statistics were calculated, and nonparametric statistical testing was performed by calculating the *p*-value of the clusters under the permutation distribution (Maris and Oostenveld, 2007), which was obtained by permuting the language labels in the original dataset 1,000 times. The sample size for these analyses was 29 participants.

Once significant clusters, i.e., time-frequency regions where neural responses to French and English are significantly different, had been identified (Figure 3C), the mean spectral amplitude in the cluster’s region was computed for each language separately. A neural measure of language discrimination was obtained by calculating the mean amplitude difference between the two language conditions (French – English) in the region of the significant cluster. This process yielded one discrimination measure per participant, which represents the candidate predictor of later language skills.

### 2.6 Predicting language outcome

Measures of language development were obtained from the CDI questionnaires collected at 12 and 18 months. Receptive vocabulary was assessed as the number of *words understood*, and expressive vocabulary was assessed as the number of *words produced* at each given age. Data from one infant were removed from analysis because expressive vocabulary at 12 months was larger than 3 SDs above the mean of the same score in the group.

**Figure 4:**
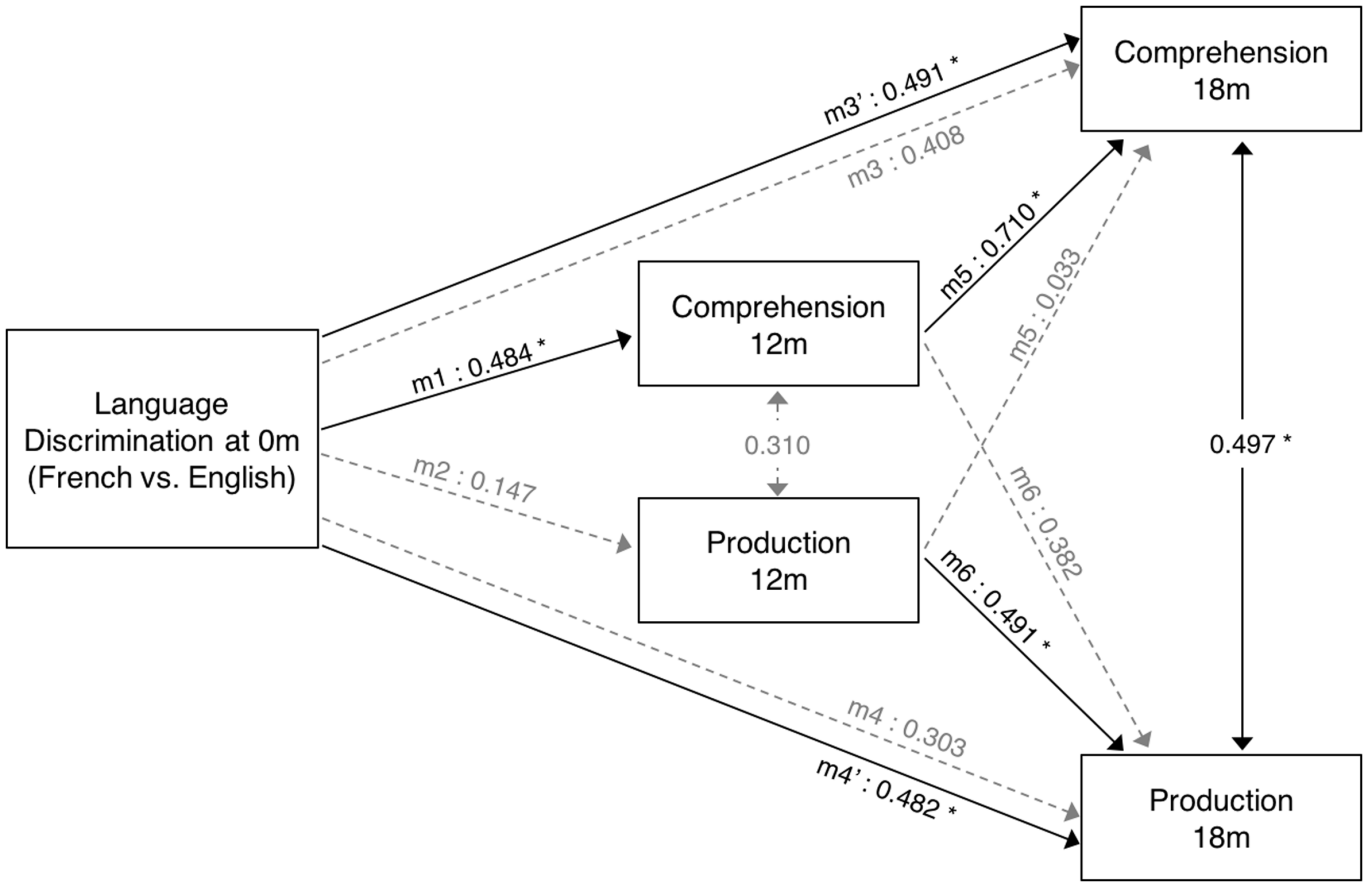
Diagram of path analysis assessing the relationship between language measures from birth to 18 months. Models 1 to 4 assess the predictive role of language discrimination at birth on vocabulary measures at 12 and 18 months. Models 5 and 6 assess the predictive relationship between vocabulary measures at 12 and 18 months. The single-ended arrows represent the predictive relationships under evaluation, and the double-ended arrows illustrate the non-causal relationships between variables (correlation). The solid black arrows illustrate significant relationships, while dashed gray arrows illustrate non-significant relationships.

To investigate the predictive role of language discriminations at birth on later language development, I conducted a path analysis considering the neural activation difference between French and English as the independent variable, and vocabulary measures at 12 and 18 months (words understood and words produced) as dependent variables. Figure 4 depicts the relationships that were assessed. Additionally, to evaluate whether CDI data reliably tracks infants’ vocabulary growth, the predictive role that vocabulary measures at 12 months have on vocabulary measures at 18 months was also evaluated. Three hypothesis were tested here: (i) neural data at birth can predict vocabulary skills at 12 months; (ii) neural data at birth can predict vocabulary skills at 18 months; (iii) vocabulary skills at 12 months can predict vocabulary skills at 18 months. Two comparisons evaluated each hypothesis: one predicting the number of words understood and another one the number of words produced. The Bonferroni correction was applied to adjust the original alpha value (*α* = 0.05) and correct for the multiple comparisons evaluating the same hypothesis (*n* = 2). This resulted in the adjusted alpha value (*α* = 0.025), which was used to evaluate the obtained results. To test for outliers, data’s residuals and influential cases were investigated. Residuals were evaluated by assessing heteroskedasticity with the White test and the Breusch-Pagan test. To identify possible influential cases, Cook’s distance and leverage values were computed.

Additionally, to assess the relationship between language skills at a given age, Pearson’s correlation coefficients (two-tailed) were computed between the number of words understood and the number of words produced at 12 and 18 months, separately. All statistical analyses were carried out with SPSS 29 (IBM).

## 3 Results

### 3.1 EEG data analysis

A time-frequency response to French and English was obtained for the 29 participants who contributed at least 20 non-rejected epochs per condition. Figure 3A presents the group mean time-frequency maps for the two conditions at channel F4. Neural activation differences between French and English were assessed by submitting their time-frequency responses to permutation testing involving paired-samples t-tests (two-tailed). Figure 3B presents the P-map for this comparison, and Figure 3C highlights the time-frequency regions where differences take place at channel F4. Supplementary Figure S4 presents the results for all channels.

A significant cluster revealing neural activation differences between French and English was found at channel F4 ranging from 4 to 5 Hz [*t* (28) = 862,17; *p* = 0.02]. In the cluster region, neural responses exhibit higher activation for French (the native language) than for English (the rhythmically different unfamiliar language), mainly at 5 Hz, during the first half of the sentences. The maximum effect size, partial eta-squared (n_p_^2^), for this significant cluster in channel F4 is 0.9794. These results were obtained for a subset of participants (*n* = 29) from the original publication (*n* = 40) investigating neural oscillations at birth (Ortiz-Barajas et al., 2023), therefore they reveal the same findings: theta activity in the human newborn brain is sensitive to rhythmic differences across languages as it can successfully distinguish between the rhythmically different languages, English, a stress-timed language, and French, a syllable-timed language (Ramus et al., 1999).

The language discrimination measure, defined as the difference in neural activation between French and English, ranged from −0.422 to 0.896 (mean = 0.204, SD = 0.298). Supplementary Table S3 presents the language discrimination measure (Discrimination_Theta_F4_0m) for the 29 included participants.

### 3.2 Predicting language outcome

Measures of language development were obtained by collecting information about children’s receptive and expressive vocabulary at 12 and 18 months. Supplementary Table S3 presents the vocabulary measures (words understood, words produced) for the 29 included participants.

A path analysis was conducted to evaluate the predictive relationship between language discrimination at birth and language skills at 12 and 18 months. Additionally, the predictive relationship between language measures at 12 and 18 months was also evaluated to assess infant’s vocabulary growth. Table 1 presents the results of the linear regression models, and Figure 4 depicts the standardized estimates of the path coefficients.

**Table 1:** Regression models assessing the prediction of language skills at 12 and 18 months.

**Model**	**Dependent variable**	***R***	***R* square**	**df**	***F***	**Sig**	**Independent variable**	**Beta**	**Sig**	**Sample size**
1	Comprehension_12m	0.484	0.234	1	6.110	0.023*	Discrimination_0m	0.484*	0.023*	22
2	Production_12m	0.147	0.022	1	0.444	0.513	Discrimination_0m	0.147	0.513	22
3	Comprehension_18m	0.408	0.167	1	4.996	0.035	Discrimination_0m	0.408	0.035	27
4	Production_18m	0.303	0.092	1	2.534	0.124	Discrimination_0m	0.303	0.124	27
5	Comprehension_18m	0.725	0.525	2	9.398	0.002*	Comprehension_12m; Production_12m	**0.710**; 0.033	0.001*; 0.859	20
6	Production_18m	0.741	0.549	2	10.366	0.001*	Comprehension_12m; Production_12m	0.382; **0.491**	0.049; 0.015*	20

Models 1 to 4 use language discrimination at birth as predictor, while models 5 and 6 explore the predictive relationship between vocabulary measures at 12 and 18 months.

The alpha value for these tests is *α* = 0.025.

**Figure 5:**
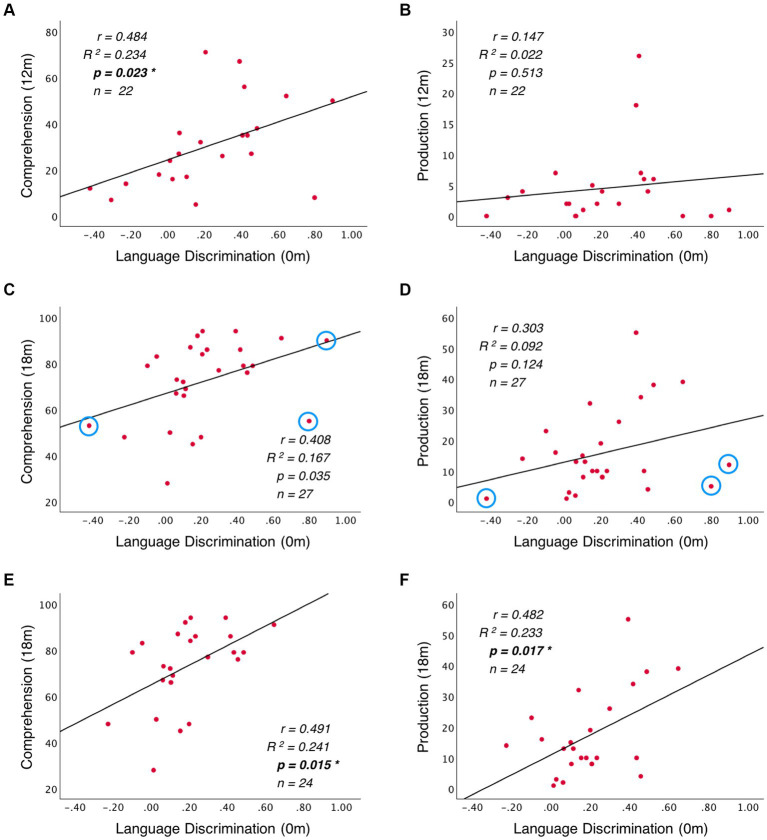
Linear regression between language discrimination at birth and: **(A)** language comprehension at 12 months, **(B)** language production at 12 months, **(C)** language comprehension at 18 months, and **(D)** language production at 18 months. Panels **(E)** and **(F)** illustrate the linear regressions from **(C)** and **(D)** respectively, after removing the outliers highlighted with blue circles.

When evaluating the predictive role of language discrimination at birth, a significant path coefficient was found for language comprehension at 12 months (Beta = 0.484, *p* = 0.023, model 1). This significant linear relationship is illustrated in Figure 5A. In contrast, language discrimination at birth did not predict production skills at 12 months (Beta = 0.147; *p* = 0.513, model 2), nor language comprehension at 18 months (Beta = 0.408; *p* = 0.035, model 3), nor language production at 18 months (Beta = 0.303; *p* = 0.124, model 4). Figures 5B–D illustrate the non-significant linear regressions evaluated for language production at 12 months, as well as for language comprehension and production at 18 months, respectively. When evaluating for outliers, the model assessing the prediction of production skills at 18 months (model 4) exhibited heteroskedasticity according to Breusch-Pagan test (Chi-Square = 4.924, *p* = 0.026). Additionally, 3 influential cases were identified in the models assessing the prediction of language skills at 18 months (models 3 and 4) due to having leverage values greater than twice the average (leverage values = 0.21, 0.16, and 0.19; average value = 0.07). Supplementary Table S3 highlights the influential cases in red, and Figures 5C,D identifies them with blue circles. As post-hoc analyses, the 3 influential cases were removed and regression models were re-calculated (Table 2, models 3′ and 4′). Language discrimination at birth was found to significantly predict language comprehension (Beta = 0.491; *p* = 0.015, model 3′) and language production (Beta = 0.482; *p* = 0.017, model 4′) at 18 months, after the 3 influential cases were removed. Figures 5E,F illustrate how these linear regressions, excluding the influential cases, predict language skills at 18 months (models 3′ and 4′).

**Table 2:** *Post-hoc* regression models.

**Model**	**Dependent variable**	***R***	***R* square**	**df**	***F***	**Sig**	**Independent variable**	**Beta**	**Sig**	**Sample size**
3′	Comprehension_18m	0.491	0.241	1	6.988	0.015*	Discrimination_0m	**0.491**	0.015*	24
4′	Production_18m	0.482	0.233	1	6.676	0.017*	Discrimination_0m	**0.482**	0.017*	24
5′	Comprehension_18m	0.724	0.524	1	19.830	<0.001*	Comprehension_12m	**0.724**	<0.001*	20
6′	Production_18m	0.656	0.431	1	13.622	0.002*	Production_12m	**0.656**	0.002*	20

Models 3′ and 4′ represent post-hoc linear regressions of models 3 and 4 (Table 1) after removing 3 influential cases.

Model 5′ and 6′ represent post-hoc linear regressions of models 5 and 6 (Table 1) after removing the non-significant predictors.

The alpha value for these tests is *α* = 0.025.

**Figure 6:**
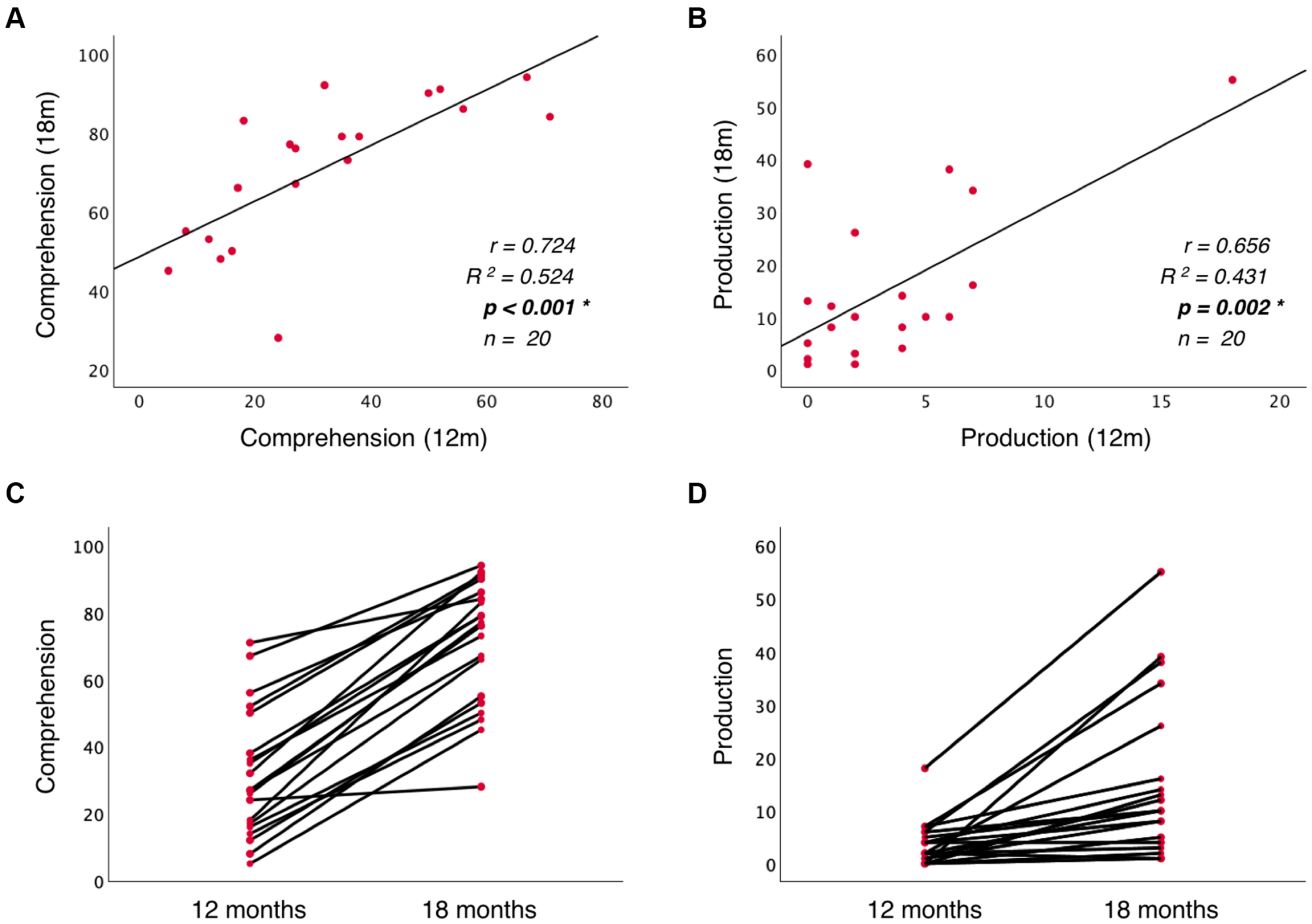
Panels **(A)** and **(B)** illustrate the linear relationship between vocabulary measures at 12 and 18 months, while **(C)** and **(D)** illustrate the trajectory of vocabulary growth from 12 to 18 months at the participant level.

To assess whether language abilities at 12 months are representative of the developmental path that language acquisition will follow, I assessed the predictive relationship between vocabulary measures at 12 and 18 months. The results show that language comprehension at 18 months is significantly predicted by language comprehension at 12 months (Beta = 0.710; *p* = 0.001, model 5), but not by language production at 12 months (Beta = 0.033; *p* = 0.859, model 5). Similarly, language production at 18 months is significantly predicted by language production at 12 months (Beta = 0.491; *p* = 0.015, model 6), but not by language comprehension at 12 months (Beta = 0.382; *p* = 0.049, model 6). Table 2 presents post-hoc regression analyses (models 5′ and 6′) removing the non-significant predictors from models 5 and 6 (Table 1). Figures 6A,B illustrate the significant linear relationship between vocabulary measures at 12 and 18 months. Furthermore, Figures 6C,D illustrate the developmental trajectories for word comprehension and word production respectively, exhibiting a vocabulary growth that is consistent across participants. These results confirm that CDI questionnaires provided reliable measures of language growth in this sample.

When evaluating the relationship between language skills at 12 and 18 months, a significant positive correlation was observed between language comprehension and production at 18 months (r = 0.497, *p* = 0.008, *n* = 27), but not between vocabulary measures at 12 months (*r* = 0.310, *p* = 0.160, *n* = 22). These non-predictive relationships are depicted in Figure 4 with double-sided arrows.

## 4 Discussion

The current study investigated whether a neural measure of language discrimination at birth, defined as the neural activation difference found when processing the prenatally heard language (French) and a rhythmically different unfamiliar language (English), could be used as predictor of language outcome. Results revealed that differences in theta activity at birth, claimed to reflect rhythmic discrimination of French and English predict language comprehension at 12 months. Furthermore, post-hoc analyses after removing 3 outliers from the vocabulary data at 18 months revealed that language discrimination at birth also predicts language comprehension and production at 18 months. These findings suggest that the ability to recognize the native language and discriminate it from a rhythmically different unfamiliar language at birth can predict later language development.

When newborns discriminate their native language from a rhythmically different unfamiliar language, they perform two tasks: (1) they discriminate the acoustic features that differentiate both rhythmic classes, and (2) they recognize the features of their native language (heard *in utero*). Therefore, a language discrimination task involving the native language, is different from a discrimination task involving two unfamiliar languages (Byers-Heinlein et al., 2010). Here, newborns discriminated their native language (French) from a rhythmically different unfamiliar language (English). This discrimination was reflected by activation differences in the theta band such that, at the group level, higher theta activation was exhibited for French that for English. Such activation differences could have been originated from different activation profiles: (i) activation for French and no activation for English, (ii) no activation for French and suppression for English, or (iii) activation for French and English, with higher activation for French. Findings from my previous study investigating neural oscillations during speech processing at birth, using a superset of the current dataset (Ortiz-Barajas et al., 2023), revealed that theta activity during French and English processing was higher than at rest, pointing in the direction of situation (iii), where activation for both languages takes place, and differences originate from higher activation to French. This supports the hypothesis that the modulation of theta activity might be one way for the newborn brain to encode speech rhythm (regardless of language familiarity), aiding in the discrimination of rhythmically different languages, and the recognition of the native language (Ortiz Barajas et al., 2021; Ortiz Barajas and Gervain, 2021; Ortiz-Barajas et al., 2023).

Furthermore, theta activity in the newborn brain has also been found to exhibit increased long-range temporal correlations after stimulation with the prenatally heard language, indicating the early emergence of brain specialization for the native language (Mariani et al., 2023). If stronger theta activation for French (as compared to English) reflects brain specialization for the native language, a discrimination measure reflecting this activation difference should predict infants’ later language abilities. Results from the current study revealed that larger discrimination measures at birth predict higher vocabulary measures at 12 and 18 months, while lower discrimination measures predict lower later language skills. These findings suggest that language discrimination at birth represents an early measure of neural commitment to the native language that predicts its later developmental trajectory. Theta activity has been argued to support the processing of syllabic units in adults (Ghitza and Greenberg, 2009). Findings from infant studies point in the same direction, as theta activity has been found to underlie language discrimination (Nacar Garcia et al., 2018; Ortiz-Barajas et al., 2023), suggesting that it might encode speech rhythm. Additionally, theta activity in the infant brain has also been found to synchronize to the speech envelope (Ortiz Barajas et al., 2021), and the speech envelope carries rhythm (Rosen, 1992). Since both the speech envelope and rhythm correlate with syllabic rate (Varnet et al., 2017; Zhang et al., 2023), it is reasonable to suggest that theta activity might encode syllabic units, and rhythm, by extracting relevant features from the speech envelope already at birth. If this is the case, the predictive power of the language discrimination measure at birth could be due to theta activity favoring the encoding of syllables in French (a syllable-timed language), which in turn would favor later word learning. This claim is supported by previous studies showing that tracking of stressed syllables at 10 month (Menn et al., 2022) and learning of disyllabic words at birth (Suppanen et al., 2022) predict language abilities at 2 years. These results taken together suggest that syllable encoding supports word-segmentation and word learning, which in turn support language development. Newborns have been shown to have a universal sensitivity to syllables (Sansavini et al., 1997; Ortiz Barajas and Gervain, 2021), however, it cannot be established whether the larger theta activity observed here on prenatally French-exposed newborns reflects good encoding of syllabic units due to this inherent (universal) ability, or whether prenatal experience with French (a syllable-timed language) has strengthened this sensitivity. Future research testing the same stimuli on prenatally English-exposed newborns (English being a stress-timed language) will shed light on the role of theta activity on syllable encoding at birth.

When exploring the predictive role of language discrimination at birth on later language skills, a significant linear relationship was found with language comprehension at 12 months, as well as with language comprehension and production at 18 months (after removing outliers). These results (Figure 4) depict a language trajectory that is coherent and consistent along development: language scores at any given age predict language scores at a subsequent age. However, one exception was found for language production at 12 months, which was not predicted by language discrimination at birth. This could be because at 12 months, language production is at its very beginning (Figure 2) and individual variability is low (Figures 5B, 6D). This suggests that measuring language production at 12 months is too early to describe the language developmental trajectory of each individual. This is supported by the fact that language production at 12 months is not correlated with language comprehension at the same age, which on the contrary, does describe the language trajectory of participants. However, language production undergoes an accelerated growth around 18 months (vocabulary spurt) (Kuhl, 2007), and becomes a better indicator of the language trajectory, as it correlates with language comprehension at the same age, and it can be predicted by language discrimination at birth.

In summary, the current study revealed a predictive relationship between a measure of theta activity during language discrimination at birth and later language outcome that merits further exploration and confirmation in future studies. These results point toward a developmental scenario in accordance with theoretical predictions as well as empirical findings: prenatal experience with speech mainly consists of language prosody, as maternal tissues filter out the higher frequencies, but preserve the low-frequency components that carry prosody (Pujol et al., 1991). Having experience with the prosody of their mother’s language, allows newborns to identify it and discriminate it from other rhythmically different languages at birth. Low frequency neural activity (delta and theta) has been found to support speech processing at birth, and to reflect rhythmic language discrimination, suggesting that it reflects the processing of prosody (Ortiz-Barajas et al., 2023). Considering the relevance of low frequency neural activity in speech processing at birth, as well as in adulthood (Giraud and Poeppel, 2012; Meyer, 2018), it is reasonable to hypothesize that it has a central role in language acquisition, as not only it describes speech processing at the time of measurement, it also seems to describe the language developmental trajectory a child might follow.

## Data availability statement

The processed EEG data that support the findings of this study have been deposited in the OSF repository https://osf.io/4w69p.

## Ethics statement

The studies involving humans were approved by the CER Paris Descartes ethics committee of the Paris Descartes University (currently, Université Paris Cité). The studies were conducted in accordance with the local legislation and institutional requirements. Written informed consent for participation in this study was provided by the participants’ legal guardians/next of kin.

## Author contributions

MCO-B: Investigation, Formal analysis, Writing – original draft, Writing – review & editing.
