# Supplementary material for: Predicting language outcome at birth
Source: Front Hum Neurosci. 2024 Jul 5;18:1370572. doi: 10.3389/fnhum.2024.1370572 (PMC11258996; doi:10.3389/fnhum.2024.1370572)
Supplement: Supplementary file 1 [file Data_Sheet_1.pdf]

Supplementary Material

Predicting Language Outcome at Birth

Maria Clemencia Ortiz-Barajas *

*** Correspondence: Maria Clemencia Ortiz-Barajas**: mariac.ortizb@gmail.com

Baby	EEG_0m	French	English	CDI_12m	CDI_18m
1	X	o	o	X	X
2	X	o	o		X
3	X	o	o	X	X
4	X	o	o	X	X
5	X	o	o	X	X
6	X	o	o	X	X
7	X	o	o	X	X
8	X	o	o	X	X
9	X	o	o		X
10	X	o	o	X	X
11	X			X	X
12	X	o	o	X	X
13	X	o	o		X
14	X	o		X	
15	X	o	o	X	
16	X	o	o	X	X
17	X	o	o	X	X
18	X	o	o	X	
19	X	o	o	X	X
20	X	o	o	X	X
21	X			X	
22	X	o	o	X	X
23	X	o		X	X
24	X	o	o		X
25	X	o	o		X
26	X	o	o		X
27	X	o	o	X	X
28	X	o	o		X
29	X	o	o	X	X
30	X	o	o	X	X
31	X	o	o	X	X
32	X		o		X
33	X	o	o	X	X
34	X	o	o	X	X
35	X		o	X	

Supplementary Table 1. Summary of data points collected for each participant longitudinally. An "X" indicates the contribution of data at a given age, and an "o" indicates the availability of good quality EEG data for a given language condition. A total of 35 participants contributed EEG data at birth (EEG_0m) and with at least one CDI questionnaire (at 12 and/or 18 months). A subset of 29 participants contributed good quality EEG data for both language conditions of interest (French and English). CDI data was collected from 27 participants at 12 months, and 30 participants at 18 months (22 participants contributed both vocabulary assessments). A subset of 22 participants contributed good quality EEG data at birth and CDI data at 12 months, while 27 participants contributed good quality EEG data at birth and CDI data at 18 months.

	Set 1	Set 2	Set 3
**English**	*The bears lived all together in a beautiful house*<br><br>Syllables: 13<br>Duration: 2,50 s<br>Syllabic rate: 5 Hz<br>Pitch_min: 142,5 Hz<br>Pitch_max: 700,8 Hz<br>Pitch_range: 558,3 Hz<br>Pitch_mean: 303,4 Hz	The bears decided to take a walk that day<br><br>Syllables: 11<br>Duration: 2,16 s<br>Syllabic rate: 5 Hz<br>Pitch_min: 181,2 Hz<br>Pitch_max: 400,2 Hz<br>Pitch_range: 219,0 Hz<br>Pitch_mean: 261,8 Hz	The little bears saw that their chairs were used by the naughty girl<br><br>Syllables: 15<br>Duration: 2,96 s<br>Syllabic rate: 4 Hz<br>Pitch_min: 171,2 Hz<br>Pitch_max: 735,9 Hz<br>Pitch_range: 564,7 Hz<br>Pitch_mean: 322,1 Hz
**French**	Les ours habitaient tous ensemble dans une maison<br><br>Syllables: 13<br>Duration: 2,66 s<br>Syllabic rate: 4 Hz<br>Pitch_min: 195,6 Hz<br>Pitch_max: 556,5 Hz<br>Pitch_range: 360,9 Hz<br>Pitch_mean: 307,6 Hz	Les ours décidèrent d'aller se promener<br><br>Syllables: 11<br>Duration: 2,17 s<br>Syllabic rate: 4 Hz<br>Pitch_min: 99,7 Hz<br>Pitch_max: 342,3 Hz<br>Pitch_range: 242,6 Hz<br>Pitch_mean: 224,7 Hz	Les ours virent que leurs chaises avaient été utilisées<br><br>Syllables: 15<br>Duration: 2,93 s<br>Syllabic rate: 6 Hz<br>Pitch_min: 95,7 Hz<br>Pitch_max: 263,2 Hz<br>Pitch_range: 167,5 Hz<br>Pitch_mean: 190,1 Hz
**Spanish**	Los osos vivían juntos en una casa<br><br>Syllables: 13<br>Duration: 2,60 s<br>Syllabic rate: 4 Hz<br>Pitch_min: 164,4 Hz<br>Pitch_max: 287,3 Hz<br>Pitch_range: 122,9 Hz<br>Pitch_mean: 219,8 Hz	Los osos tomaron un gran paseo<br><br>Syllables: 11<br>Duration: 2,19 s<br>Syllabic rate: 3 Hz<br>Pitch_min: 178,2 Hz<br>Pitch_max: 423,5 Hz<br>Pitch_range: 245,3 Hz<br>Pitch_mean: 245,9 Hz	Los osos vieron sus sillas siendo utilizadas<br><br>Syllables: 15<br>Duration: 3,00 s<br>Syllabic rate: 4 Hz<br>Pitch_min: 99,0 Hz<br>Pitch_max: 420,8 Hz<br>Pitch_range: 321,8 Hz<br>Pitch_mean: 260,0 Hz

Supplementary Table 2. Stimulus information. Three sets of sentences were used during the study. Each set contained one sentence translated into the three languages. The translations were slightly modified in order to match sentence duration across languages within the same set. The duration, syllabic rate, and pitch details are included for each sentence. Table taken from Ortiz Barajas et al., (2021).

Baby	Discrimination_Theta_F4 (0m)	words_understood (12m)	words_produced (12m)	words_understood (18m)	words_produced (18m)
1	0,1789	32	2	92	10
2	0,2076			94	8
3	-0,2267	14	4	48	14
4	0,8962	50	1	90	12
5	0,0126	24	2	28	1
6	0,4551	27	4	76	4
7	0,3910	67	18	94	55
8	0,6450	52	0	91	39
9	0,1989			48	19
10	0,1530	5	5	45	10
11	0,2058	71	4	84	8
12	0,1131			69	13
13	0,4067	35	26		
14	0,0642	36	0	73	13
15	0,4341	35	6	79	10
16	-0,3065	7	3		
17	0,4167	56	7	86	34
18	-0,0470	18	7	83	16
19	0,7990	8	0	55	5
20	0,2324			86	10
21	0,0998			72	15
22	-0,0988			79	23
23	0,2976	26	2	77	26
24	0,1398			87	32
25	-0,4220	12	0	53	1
26	0,4856	38	6	79	38
27	0,0607	27	0	67	2
28	0,1035	17	1	66	8
29	0,0267	16	2	50	3

Supplementary Table 3. Summary of measures used to predict language outcome. A total of 29 participants contributed a language discrimination measure at birth (Discrimination_Theta_F4_0m). A subset of 22 participants contributed vocabulary measures at 12 months (words_understood_12m, words_produced_12m), while 27 participants contributed vocabulary measures at 18 months (words_understood_18m, words_produced_18m). Values highlighted in red were identified as influential cases in models 3 and 4 due to having leverage values greater than twice the average (leverage values = 0.21, 0.16, and 0.19; average value = 0.07).

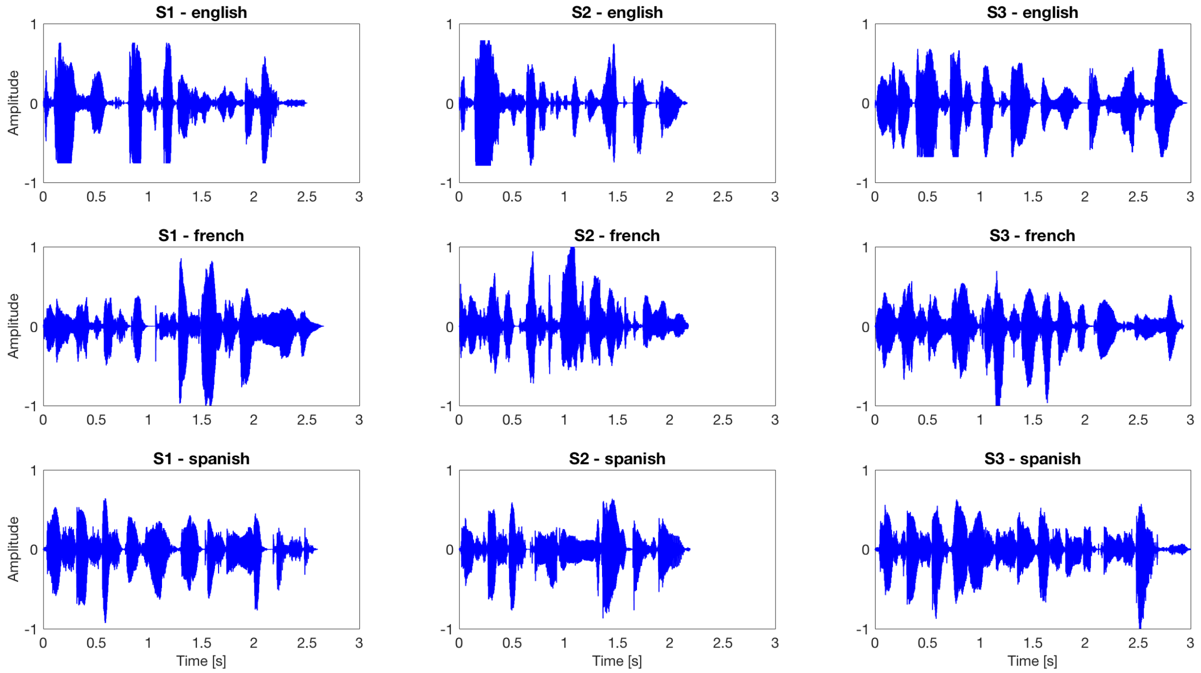

Supplementary Figure 1. Time series for the nine utterances used as stimuli. Figure taken from Ortiz-Barajas et al., (2023).

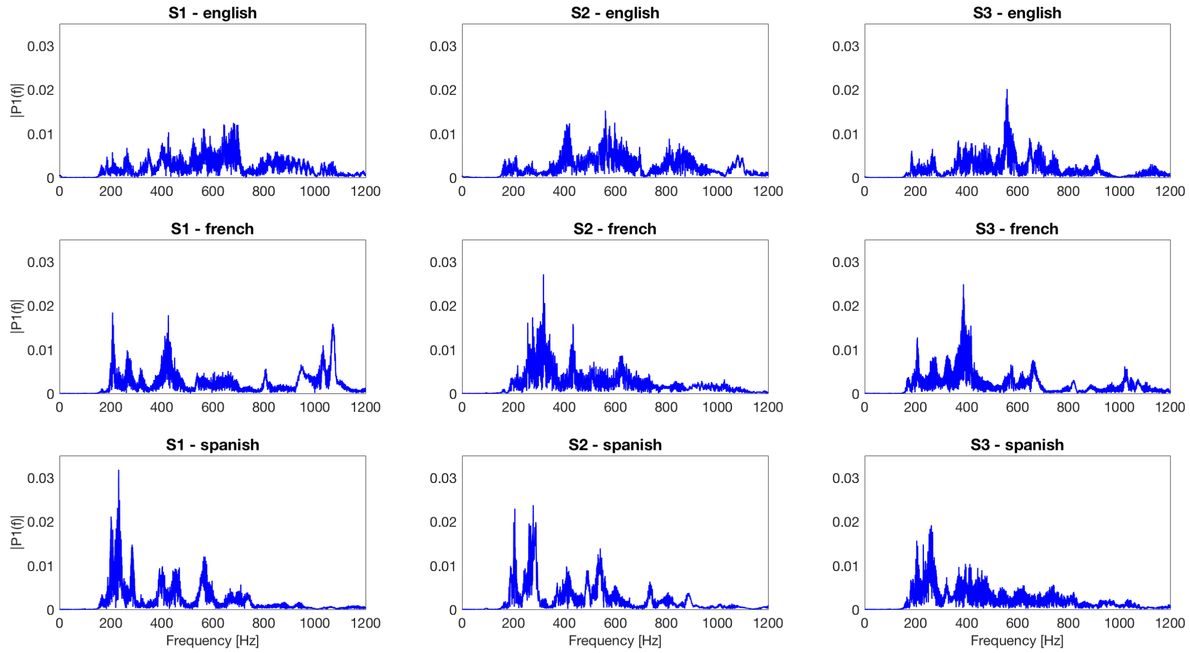

Supplementary Figure 2. Frequency spectra for the nine utterances used as stimuli. These frequency spectra were obtained using the Fast Fourier Transform function in Matlab (fft). Figure taken from Ortiz-Barajas et al., (2023).

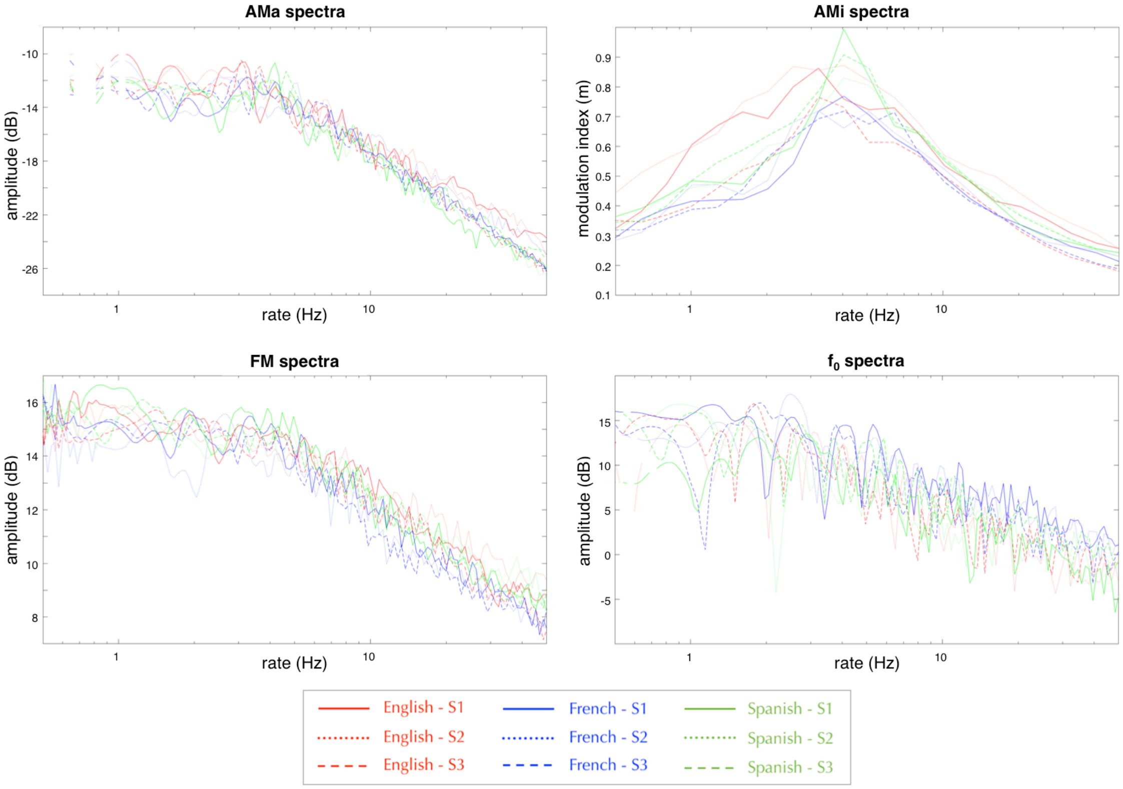

Supplementary Figure 3. Amplitude and frequency modulation spectra for the nine utterances used as stimuli. Red lines: English utterances; blue lines: French utterances; green lines: Spanish utterances. These modulation spectra were obtained following the methodology described by Varnet and colleagues (Varnet et al., 2017). Figure taken from Ortiz Barajas et al., (2021).

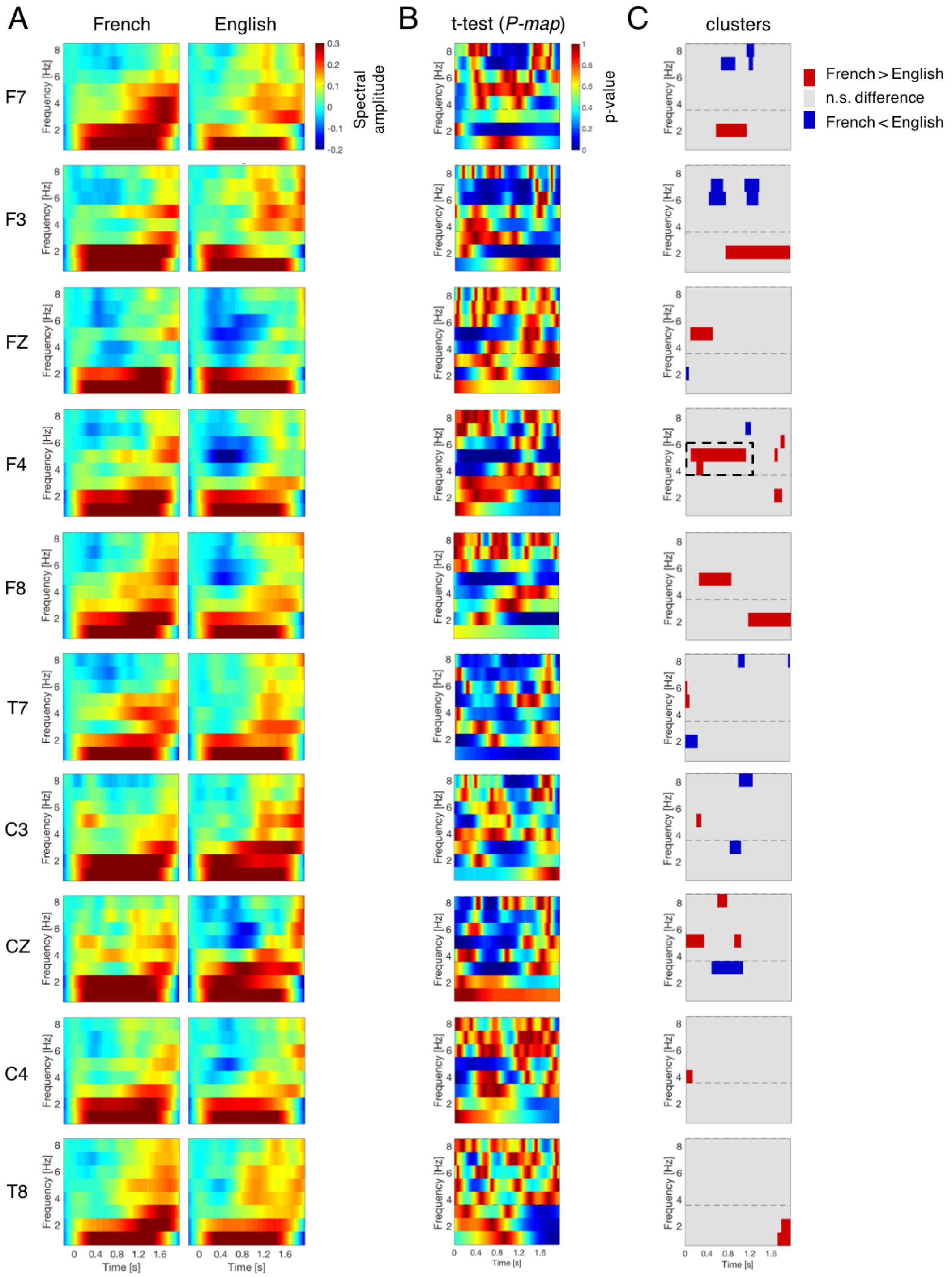

Supplementary Figure 4. Neural activation during speech processing at birth. (A) Average time-frequency response to French and English per channel. The time-frequency maps illustrate the mean spectral amplitude per condition from 1 to 8 Hz. (B) P-maps obtained by submitting the time-frequency responses to French and English to paired-samples t-tests (two-tailed). (C) Time-frequency regions where the absolute T-values exceed the critical threshold (|T-value| > 2.048). Red regions indicate higher activation for French, while blue regions indicate higher activation for English. The dashed rectangular box at channel F4 indicates the cluster exhibiting significant differences between French and English.

References

Ortiz Barajas, M. C., Guevara, R., & Gervain, J. (2021). The origins and development of speech envelope tracking during the first months of life. *Developmental Cognitive Neuroscience*, *48*, 100915. https://doi.org/10.1016/j.dcn.2021.100915

Ortiz-Barajas, M. C., Guevara, R., & Gervain, J. (2023). Neural oscillations and speech processing at birth. *iScience*, *26*(11), 108187. https://doi.org/10.1016/j.isci.2023.108187

Varnet, L., Ortiz-Barajas, M. C., Erra, R. G., Gervain, J., & Lorenzi, C. (2017). A cross-linguistic study of speech modulation spectra. *The Journal of the Acoustical Society of America*, *142*(4), 1976–1989. https://doi.org/10.1121/1.5006179
